# MMP‐7, ‐8, ‐9, E‐cadherin, and beta‐catenin expression in 34 ameloblastoma cases

**DOI:** 10.1002/cre2.331

**Published:** 2020-09-28

**Authors:** Jetta Kelppe, Hanna Thorén, Caj Haglund, Timo Sorsa, Jaana Hagström

**Affiliations:** ^1^ Department of Pathology, Haartman Institute University of Helsinki and HUSLAB Helsinki Finland; ^2^ Department of Oral and Maxillofacial Surgery, Institute of Dentistry University of Turku Turku Finland; ^3^ Department of Oral and Maxillofacial Diseases Turku University Hospital Turku Finland; ^4^ Department of Surgery University of Helsinki and Helsinki University Hospital Helsinki Finland; ^5^ Research Programs Unit, Translational Cancer Medicine University of Helsinki Helsinki Finland; ^6^ Department of Oral and Maxillofacial Diseases, Head and Neck Centre University of Helsinki and Helsinki University Hospital Helsinki Finland; ^7^ Department of Dental Medicine Karolinska Institute Huddinge Sweden; ^8^ Department of Oral Pathology and Radiology University of Turku Turku Finland

**Keywords:** ameloblastoma, beta‐catenin, E‐cadherin, MMP‐7, MMP‐8, MMP‐9

## Abstract

**Objectives:**

Ameloblastoma is a benign, locally aggressive odontogenic tumor with high recurrence rates. Matrix metalloproteinases (MMPs) mediate extracellular integrity in normal and pathological conditions, and exert multiple functions coordinating inflammation and tumor progression. E‐cadherin and beta‐catenin are adherence junction molecules in cell‐to‐cell connections. We investigated the involvement of MMP‐7, ‐8, ‐9, E‐cadherin, and beta‐catenin in ameloblastoma and the surrounding extracellular matrix.

**Material and methods:**

Our material consisted of 30–34 tissue samples from ameloblastoma patients of Helsinki University Hospital. We used immunohistochemistry to detect the expression of the biomarkers. Two oral pathologists independently scored the immunoexpression intensities and statistical calculations were made based on the results.

**Results:**

E‐cadherin expression was weaker in the maxillary than in mandibular ameloblastomas. Beta‐catenin was expressed in the ameloblastoma cell membranes. We detected MMP‐8 and ‐9 expression in polymorphonuclear neutrophils in the extracellular area and these MMPs correlated positively with each other. Osteoclasts lining bone margins and multinuclear giant cells expressed MMP‐9. Neither MMP‐8 nor MMP‐9 immunoexpression could be detected in ameloblastoma cells. MMP‐7 expression was seen in some apoptotic cells.

**Conclusion:**

The fact that E‐cadherin immunoexpression was weaker in maxillary compared to mandibular ameloblastomas might associate to earlier recurrences. It promotes the idea of mandibular and maxillary ameloblastoma exerting differences in their biologies. We detected MMP‐8 and ‐9 in polymorphonuclear neutrophils which relates to these MMPs participating in extracellular remodeling through a mild inflammatory process. Bone degradation around ameloblastoma may be due to MMP‐9 in osteoclasts but this phenomenon might be an independent process and needs further investigations.

## 1 INTRODUCTION

Ameloblastomas are the most common odontogenic tumors with an estimated annual incidence of 0.5/million inhabitants. They are benign but locally aggressive odontogenic epithelial tumors located to the dentoalveolar region presenting as an intraosseous or peripheral lesion. Patients are typically 30–40 years old, though tumors occur in all age groups (El‐Naggar, Chan, Grandis, Takata, & Slootweg, 2017). Recurrences develop in 20–93% of cases supposedly depending on the treatment modality (Neagu, et al., 2019). Pathogenesis is currently based on BRAF and SMO mutations in the mitogen‐activated protein kinase (MAPK) and the Sonic Hedgehog signaling (SSH) pathways, respectively (Sweeney, et al., 2014). A growing interest focuses on the extracellular functions and inflammatory activities of the tumor environment. Still, the exact tumorigenesis remains to be resolved.

Matrix metalloproteinases (MMP) are a heterogeneous group of zinc‐dependent, genetically distinct but structurally related proteinases responsible for the degradation and synthesis control of the extracellular matrix (ECM) and the basement membrane (BM). MMP's also participate by processing nonmatrix bioactive substrates involved in the membrane shedding, chemokine, or growth factor modification, and in regulating the activity of other proteases. They play an important role as the effective regulators of cell proliferation and differentiation, tissue homeostasis, and immune response (Löffek, Schilling, & Franzke, 2011).

The gelatinase MMP‐9, when activated can break down type IV collagen and gelatin, the main elements of the ECM and BM (Roomi, Monterrey, Kalinovsky, Rath, & Niedzwiecki, 2009). MMP‐9 plays a crucial role in tumor progression, from angiogenesis, to stromal and bone remodeling, and ultimately to metastasis (Farina & Mackay, 2014). Various studies confirm and evidence the presence of MMP‐9 in ameloblastoma cells and the ECM and BM processings (Anne, Krisnuhoni, Chotimah, & Latief, 2014; Kumamoto, Yamauchi, Yoshida, & Ooya, 2003; Ribeiro et al., 2009; Yang, et al., 2018).

MMP‐8 or collagenase‐2 is a neutrophil derived collagenase with a multifunctional role in mediating inflammation, and inhibiting cancer invasion and metastasis. Its protective nature seems to depend on the tissue of origin (Juurikka, Butler, Salo, Nyberg, & Åström, 2019). It degrades efficiently type 1 collagen among other ECM and non‐ECM substrates. MMP‐8 is thought to play a role in inflammation and different tumor processes (Juurikka et al., 2019). To our knowledge, there is no previous studies of the MMP‐8 expression in ameloblastomas.

MMP‐7, known as matrilysin, is found constitutively in many epithelial cell types, especially ductal cells of exocrine glands like salivary glands, liver, breast and colon (Saarialho‐Kere, Crouch, & Parks, 1995). It has a function in tumor invasion, metastasis and as a pro‐MMP‐2, ‐8 and ‐9 activator. It has also been associated to angiogenesis in normal physiological processes as well as in cancer progression (Nishizuka et al., 2001)

**FIGURE 1:**
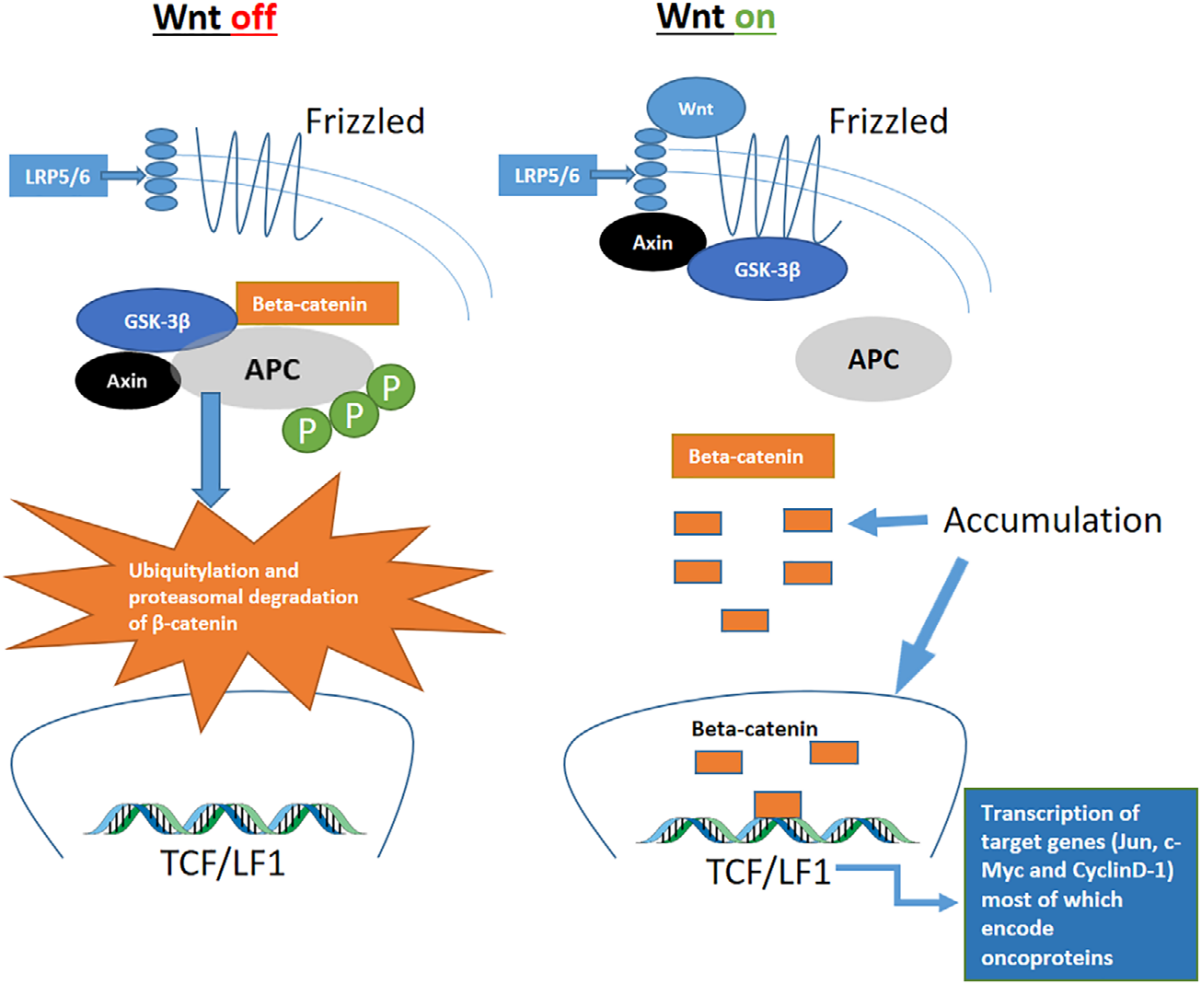
The canonical Wnt/beta‐catenin signaling pathway. Accumulation of beta‐catenin into the cytoplasm or nucleus of the cell helps maintain the stemness of cells, prompts tumorigenic qualities, and enhances cancer cell proliferation and survival. Beta‐catenin activity is controlled by numerous binding partners that affect stability, cellular localization, and transcriptional activity. Modified from Shang et al. (2017). APC, adenomatous polyposis coli; Axin, scaffold protein; GSK3, glycogen synthase kinase 3; LPR5/6, lipopolysaccharide like receptor protein 5/6; P, phosphorylation; TCF/LEF, T‐cell factor/lymphoid enhancing factor

Adherens junctions anchor epithelial cells together and mediate cell and tissue behavior via transmembrane cadherin/catenin‐based complexes bound to intracellular microfilaments. These structures contribute to the formation of solid tissue and coordinate intra‐ and intercellular signaling (Niessen, 2007). E‐cadherin is a Ca‐dependent transmembrane protein which is linked intracellularly to p120‐, *α*‐, *β*‐, and *γ*‐catenins (Tian, Liu, Niu, et al., 2011). E‐cadherin/beta‐catenin complexes modulate Wnt signaling and are involved in epithelial to mesenchymal (EMT) and mesenchymal to epithelial (MET) transitions, which are essential in embryo development, tissue fibrosis, and cancer progression. Mediators of inflammation, including MMPs, growth factors, and cytokines may cause dysregulation and loosening of this adherence complex (Shang, Hua, & Hu, 2017). Via transactivation of target genes involved, the nuclear accumulation of beta‐catenin promotes tumor progression and proliferation (Brabletz, Jung, Dag, Hlubek, & Kirchner, 1999) (Figure 1). Beta‐catenin can regulate the expression of the MMP‐7 in human colorectal cancer (Brabletz et al., 1999).

In this study, we explored the expression of MMP‐7, ‐8, ‐9, beta‐catenin, E‐cadherin in the ECM and ameloblastoma tumor cells and evaluated the role of those markers combined with clinical factors in predicting recurrence of ameloblastoma.

## 2 MATERIAL AND METHODS

### 2.1 Patient material

Our series consisted of 34 ameloblastoma patients treated at the Helsinki University Hospital, Department of Oral and Maxillofacial Diseases (HUCH). The Department of Pathology (HUSLAB) archives provided us with the formalin‐fixed paraffin‐embedded tissue samples. We have described the patient material in more detail in our previous study (Kelppe, et al., 2019). Since some samples were no longer available, 8 cases with recurrent ameloblastoma of which we had no primary tumor tissue available for our preliminary studies were added to the series. The Ethics Committee of Surgery and HUCH's Internal Review Board (Dnro 151/13/03/02/2015) granted their approval for this study.

### 2.2 Immunohistochemistry

For immunohistochemistry, we used 3 μm thick formalin‐fixed paraffin‐embedded tissue sections, which we attached on the glass in 60°C for 1–2 hr. The samples underwent deparaffinization in xylene. A graded alcohol series to water rehydrated the tissue. A heated buffer (Dako ENvision Flex) specific for each antibody functioned as a heat induced epitope retrieval. For staining, we used Autostainer 480 (Labvision UK Ltd., Suffolk, UK) with Dako REAL EnVision Detection System, Peroxidase, Rabbit/Mouse (Dako, Glostrup, Denmark). The primary incubation of each antibody was for 1 hr in +4°C and a secondary HRP‐coupled antibody for half an hour. To visualize results we used either a DAB (brown) or magenta (pink) chromogen. A hematoxylin incubation for 2 minutes counterstained the nuclei. A graded alcohol series dehydrated the tissue. The samples were finally mounted. Primary antibodies were: anti‐MMP‐7 (1:1000, EMD Millipore Corporation, Temecula, CA), anti‐MMP‐8 (1:400), anti‐ MMP‐9 (1:1000, NeoMarkers, Fremont CA and Calbiochem Inc., San Diego, CA), antibeta‐catenin (1:400, Thermo Fisher Scientific MA), and ant‐E‐cadherin (1:100 Thermo Fisher Scientific MA). Colon and oral squamous cell cancer tissues functioned as positive controls. For the negative control, we did not add the primary antibody.

### 2.3 Scoring of immunohistochemistry

**FIGURE 2:**
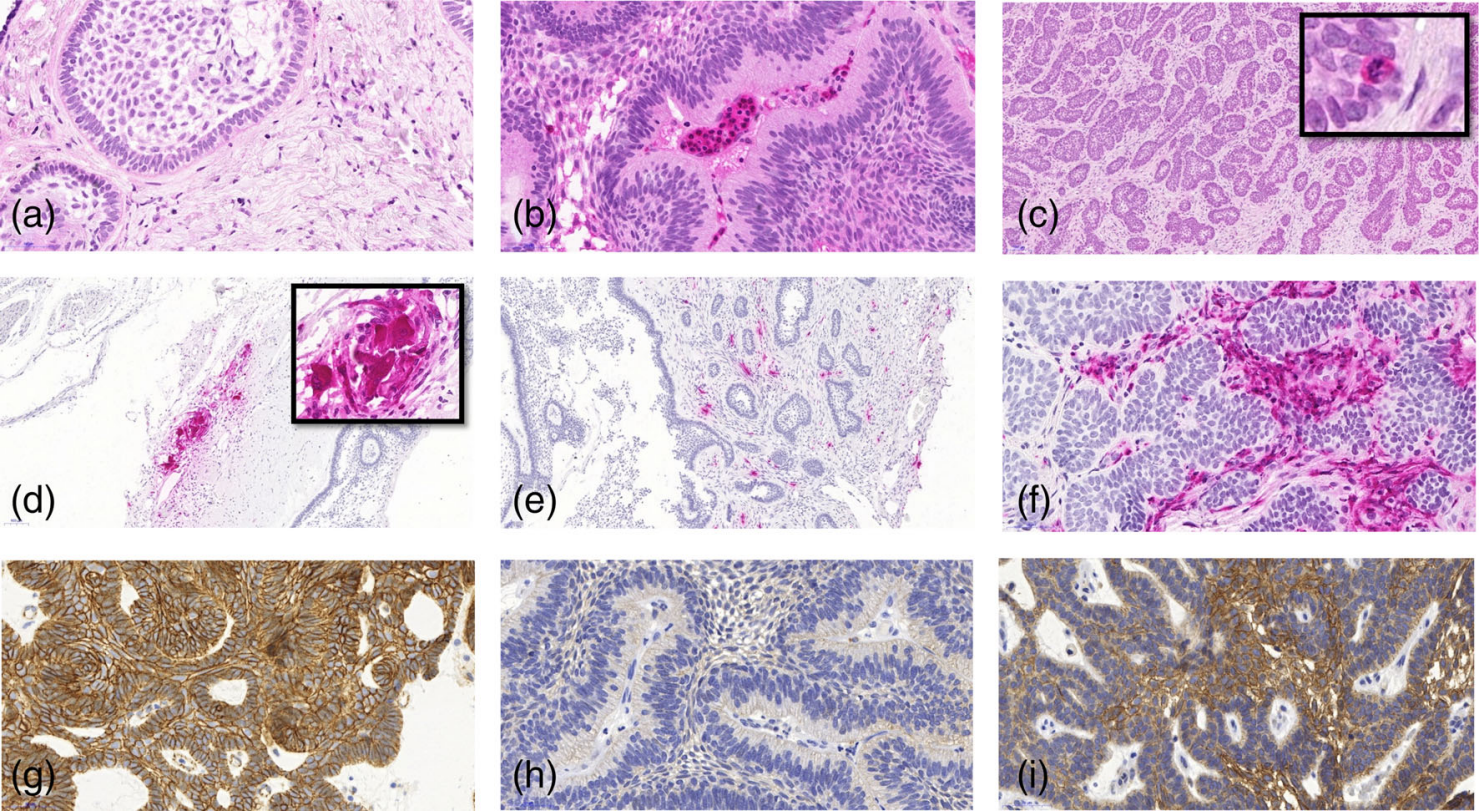
Demonstrates immunoexpression of MMP‐8, ‐7, ‐9, beta‐catenin, and E‐cadherin. Tumor cells did not express MMP‐8 (a, b). Neutrophils expressed MMP‐8‐positivity presented here as magenta red (a, b). Single apoptotic tumor cells expressed MMP‐7 (arrows) (c). Osteoclasts and fibroblasts in the invasive front expressed MMP‐9, while tumor cells remained negative (d‐f). All ameloblastomas expressed beta‐catenin (g). Ameloblastoma considered negative for E‐cadherin (h) and positive for E‐cadherin (j)

Two oral pathologists (J.K. and J.H.) scored independently the staining expressions without the knowledge of clinical data. All samples were preliminarily screened to get an overview of intensity differences in expression. We used a semiquantitative scoring system with the following guidelines: 0 when no expression was present, 1 when we saw weak reaction or only focal intense reaction, 2 when moderate diffuse reaction was seen, and 3 when intense diffuse reaction was seen. In case of disagreement, we agreed upon a consensus. MMP‐8 and MMP‐9 were scored as 0 = negative, 1 = mild positivity, 2 = strong positivity. We scored MMP7 expression negative or positive since positive cells were scarce. All tumors had a similar beta‐catenin staining pattern and were scored either 1 = mild positivity, 2 = moderately positive, or 3 = strongly positive. We scored E‐cadherin likewise as either mild or strong. Positivity less than 10% was considered as weak expression. Figure 2 shows examples of expression intensities.

### 2.4 Statistical analyzes

We sought correlations between MMP‐7, ‐8, ‐9, beta‐catenin, E‐cadherin, age, gender, location, and recurrence. We calculated odds ratios for MMP‐8 and ‐9, MMP‐8/ ‐9/beta‐catenin/and gender, recurrence, and location, MMP‐8/beta‐catenin and age. Scoring results 0 or 1 were relabeled as mild (0) and 2 or 3, were relabeled as strong (1). We used logistic regression, chi‐square tests, and when relevant, 2 by 2 table functions. We conducted the analyses using R 3.4.2 and RStudio 1.1.383 and considered a *p*‐value equal or less than .05 significant.

## 3 RESULTS

**FIGURE 3:**
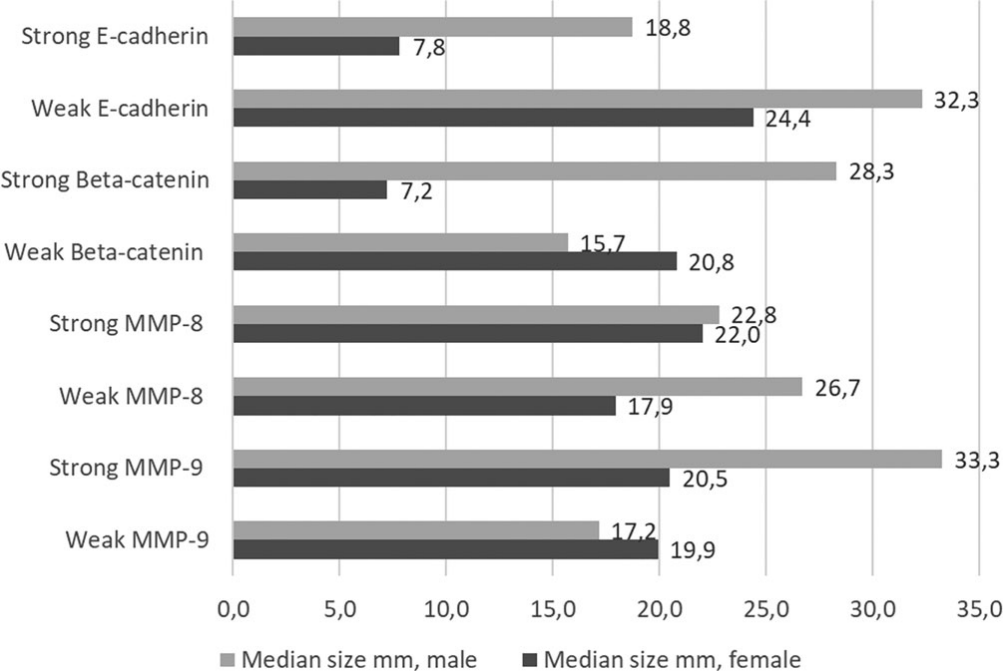
Demonstrates the difference in tumor size (median in mm) compared to E‐cadherin, Beta‐catenin, MMP‐8, and ‐9

The cohort consisted of 34 patients of which 19 were men and 15 women. The age distribution among men was 13–87 and among women 18–71. Of 34 cases, 24 were mandibular and 10 were maxillary tumors or from the sinonasal area. The tumor size varied from 7 to 110 mm. Table 1 demonstrates staining results between genders. Figure 3 shows the expression of MMPs, beta‐catenin and E‐cadherin according to size and gender. In male patients, larger tumors seemed to have decreased expression of E‐cadherin and enhanced MMP‐9 expression. In female patients E‐cadherin and beta‐catenin expression was stronger in smaller than in larger tumors (Figure 3) but without statistical significance.

**TABLE 1:** Staining results of MMP‐8, ‐9, Beta‐catenin, and E‐cadherin compared between female and male

	MMP‐8 (*n* = 34)		MMP‐9 (*n* = 34)		Beta‐catenin (*n* = 34)		E‐cadherin (*n* = 30)	
Gender	Female	Male	Female	Male	Female	Male	Female	Male
Non orweak expression	12 (35%)	1 (32%)	10 (29%)	11 (32%)	11(32%)	6 (18%)	4 (13%)	6 (20%)
Strong expression	3 (9%)	8 (24%)	5 (15%)	8 (24%)	4 (12%)	13 (38%)	8 (27%)	12 (40%)

### 3.1 MMP‐7

MMP‐7 was expressed only in apoptotic or mitotic cells in the basal layer of ameloblastoma tumor tissue which otherwise was negative. There was also a mild membranous staining. ECM was negative (Figure 2).

### 3.2 MMP‐8

Polymorphonuclear neutrophils and plasma cells showed MMP‐8 immunopositivity but ameloblastoma cells were negative (Figure 2). MMP‐8 positivity correlated with location (mandible/maxilla; *p* = .026 chi *x^2^*‐test, but when adjusted for gender and age the correlation was lost (*p =* .112).

### 3.3 MMP‐9

MMP‐9 positivity was detected in inflammatory cells, multinuclear giant cells among inflammatory infiltration, and osteoclasts lining the bone. Ameloblastoma cells were negative (Figure 2). In logistic regression, stronger MMP‐9 positivity correlated with stronger MMP‐8 (*p* = .015), but the confidence interval (CI) was wide indicating a small number of cases (OR: 8, 95% CI: 1.5, 42.4).

### 3.4 Beta‐catenin

Cell membrane expression of beta‐catenin was seen in all ameloblastoma samples, without nuclear expression. Beta‐catenin expression in ameloblastomas correlated with gender (*p* = .015), male patients having stronger expression, but the CI was once again wide (OR: 6; 95% CI: 1.3, 26.7).

### 3.5 E‐cadherin

Of 34 cases, we were able to score 30 samples for this study. Ameloblastomas expressed E‐cadherin in most of mandibular ameloblastomas (66.7%; 20/30) especially in stellate reticulum‐like areas and in peripheral columnal cells where the expression was weak or negative. Of maxillary tumors 60% (6/10) remained E‐cadherin negative or showed only weak expression (Figure 2 and Table 2). E‐cadherin expression in tumors appeared to be patchy with both positive and negative areas. Regression logistics revealed a correlation between mandibular location and strong E‐cadherin expression (*p* = .036; OR: 0.167; 95% CI: 0.031, 0.889). With logistic regression E‐cadherin correlated positively with the beta‐catenin expression (*p* = .045 with a OR: 5.4; 95% CI: 1.04, 28.5). Unfortunately, the CI was wide. Although we did not find statistical differences, the often‐recurring maxillary tumors had weak E‐cadherin expression in 4 of 6 recurring cases, whereas in recurring mandibular tumors the proportion was 1 out of 5.

**TABLE 2:** Comparison between location (mandibular or maxillary tumor), E‐cadherin staining intensity, and recurrence

	Weak E‐cadherin	Strong E‐cadherin	Total
Mandible			
No recurrence	3 (10%)	12 (40%)	15 (50%)
Recurrence	1 (3%)	4 (13.3%)	5 (16.7%)
Maxilla			
No recurrence	2 (6.7%)	2 (6.7%)	4 (13.3%)
Recurrence	4 (13.3%)	2 (6.7%)	6 (20%)
Total	10 (33.3%)	20 (66.7%)	30 (100%)

*Note*: For location and E‐cadherin expression: *p* = .045, risk ratio 2.0, 95% CI: 0.91–4.41. No significant correlations between recurrence and E‐cadherin was detected.

## 4 DISCUSSION

In this study, we used immunohistochemistry to investigate the MMP‐7, ‐8, ‐9, beta‐catenin, and E‐cadherin immunoexpression in primary and recurrent ameloblastoma tissue samples from 34 patients. In contrast to most previous studies (Anne et al., 2014; Pinheiro, Freitas, Moretti, Jorge, & Jaeger, 2004; Ribeiro et al., 2009; Souza Freitas et al., 2009; Yang et al., 2018), none of our ameloblastomas expressed MMP‐7 or ‐9. To our knowledge, there are no previous reports on MMP‐8 immunoexpression in ameloblastomas. In our study, only inflammatory cells showed MMP‐8 positivity, varying from mild to strong. Beta‐catenin was expressed on cell membranes but not, in the cytoplasm or the nucleus. Though beta‐catenin showed positivity in all ameloblastomas, E‐cadherin was unexpectedly negative in 60% of maxillary tumors. None of our parameters correlated with recurrence.

MMPs are tissue destructing proteolytic enzymes taking part in the normal and pathological growth and tissue remodeling (Uitto, Overall, & McCulloch, 2003). Different MMPs have been vastly studied in both benign, including ameloblastomas, and malignant tumors proving them to participate in tumor proliferation and progression.

Kumamoto et al. (2003) found stronger MMP‐9 positivity in ECM cells than in the epithelium of ameloblastomas, which is in line with our results. Since our preliminary staining with a Neomarks antibody presented negativity in tumor cells, we repeated the test with a Calbiochem antibody with the same negative outcome.

Ameloblastoma remodels jawbone and thins the cortical plates. Osteoclasts are known to express MMP‐9, and these cells take part in bone remodeling (Andersen, del Carmen Ovejero, Kirkegaard, Lenhard, & Foged, 2004). We found MMP‐9 positivity in multinucleated giant cells and osteoclasts as well. Otherwise, MMP‐9 was seen in inflammatory cells, that is, mainly in neutrophilic granulocytes. This could be explained by the ability of these MMPs to activate and promote inflammatory processes in the tumor microenvironment. By activating cytokines, they might take part in the tumor process as well. As mentioned in previous studies, the lack of strong immunoexpression of these ECM‐destructive enzymes in tumor tissues indicates the benign nature of ameloblastomas (Kumamoto et al., 2003; Ribeiro et al., 2009). In male patients, a more intense MMP‐9 positivity in PMN cells correlated with a stronger MMP‐8 expression. This might reflect the fact that male tumors were often larger in size, as MMP‐9 is needed for tissue destruction while the tumor is still growing, and that MMP‐8, and ‐9 may have mutual inducers in wound healing and related inflammation. Because ameloblastoma tumor tissue did not express MMP‐9 with neither of the two different MM‐9 –antibodies used, our results challenge the previous studies where MMP‐9 claimed to be expressed by the tumor cells as well (Pinheiro et al., 2004; Ribeiro et al., 2009; Yang et al., 2018). Contradictory to our results, Ribeiro et al. (2009) have demonstrated a tumorous and stromal MMP‐9 expression in ameloblastomas. These seemingly different results might be explained for example by the use of different antibody clones.

Thiolloy et al. (2009) showed that in mammary tumors osteoclast derived MMP‐7 participated in tumor induced osteolysis and tumor growth, and that MMP‐7 null mice had fewer osteoclasts at the tumor‐bone interface than did the wild‐type controls. In contrary, we could not detect MMP‐7 immunoexpression in osteoclasts. This raises the question, could the absence of osteoclast derived MMP‐7 in ameloblastomas provide protection against more aggressive behavior? In colorectal cancer, nuclear beta‐catenin can regulate MMP‐7 expression (Brabletz et al., 1999). In our material neither nuclear beta‐catenin nor MMP‐7 expression were detected in ameloblastoma cells except for some single positive apoptotic or mitotic cells.

In normal epithelial tissue, E‐cadherin/beta‐catenin structures provides a steady and adherent cell‐to‐cell junction and normal cell polarity. The loss of E‐cadherin is a principal event in metastatic progression and invasive behavior in gastric cancer (Wu, Zhuang, Jiang, et al., 2016). The downregulation of this protein might be involved in the EMT where polarized epithelial cells transform into a mesenchymal phenotype thus facilitating migration (Pasquier, Abu‐Kaoud, Al Thani, & Rafii, 2015; Shamir, et al., 2014). In addition, the accumulation of nuclear beta‐catenin activates the target genes C‐Myc, CyclinD1, MMP‐7, Twist, Snail, and Slug—all connected to EMT. In our material we could not detect MMP‐7 activation by beta‐catenin.

It has been shown that when ameloblastomas lose E‐cadherin expression they gain Snail, Slug, and Twist expression and this shift might allow tumors to gain EMT proprieties (Kurioka, Wato, Iseki, Tanaka, & Morita, 2017). We could not see morphological cell changes from epithelial to mesenchymal in our maxillary ameloblastomas, but these repressors gaining function could possibly explain the loss of E‐cadherin in these easily recurring tumors. Desmoplastic ameloblastomas differ in morphology from conventional ameloblastomas. These ameloblastoma cells are more spindle‐like and are surprisingly shown to demonstrate smooth muscle actin (SMA) positivity (Siar & Ng, 2019). In our study, most desmoplastic ameloblastoma cell strands expressed both E‐cadherin and membrane beta‐catenin showing epithelial nature. Still, the literature suggests desmoplastic ameloblastomas to potentially have aggressive biologic behavior (Sun, Wu, Cheng, Zwahlen, & Zhao, 2009). The expression of stem cell markers has been demonstrated in ameloblastomas reminding that these tumors probably possess multipotent properties enabling constant proliferation and expression of several types of molecules including p63, CD10, and the previously mentioned SMA.

We found that 60% of the maxillary tumors and 20% of the mandibular tumors had only mild E‐cadherin immunoexpression. This might reflect the fact that maxillary tumors recur more often than mandibular ones. These findings could be further investigated by staining for Vimentin, Snail, Twist, and *N*‐cadherin to exclude the possibility minor EMT‐like changes in maxillary tumors. Further research is also needed to solve the mechanism behind the weaker expression of E‐cadherin in maxillary ameloblastomas and its possible role in tumor recurrence.

In our material, ameloblastoma tissue showed solely cell membranous expression of beta‐catenin without cytoplasmic or nuclear expression. This might reflect a protective role inhibiting invasion, as nuclear beta‐catenin expression is connected to the metastatic potential (Brabletz et al., 1999). The role of beta‐catenin immunoexpression in metastatic ameloblastomas and ameloblastic carcinomas could be worth investigating.

This study would have benefited from a larger case cohort especially of maxillary tumors. Another limiting factor in this study is the semiquantitative scoring method which provides approximate estimates of the exact clinical situation. With a larger cohort and compute scoring program more precise results could be obtained.

In conclusion, to our estimate, inflammatory cells expressing MMP‐8 and ‐9 might promote tumor growth by providing extracellular degradation or cytokine activation. In maxillary tumors, the weaker expression of E‐cadherin might also facilitate tumor growth and promote recurrence.

## CONFLICT OF INTEREST

The authors do not have conflict of interest concerning this article.

## Data Availability

Research data are not shared.
